# What advice are oncologists and surgeons in the United Kingdom giving to breast cancer patients about physical activity?

**DOI:** 10.1186/1479-5868-5-46

**Published:** 2008-09-19

**Authors:** Amanda J Daley, Sarah J Bowden, Daniel W Rea, Lucinda Billingham, Amtul R Carmicheal

**Affiliations:** 1 Primary Care Clinical Sciences, School of Health and Population Sciences, College of Medical and Dental Sciences, University of Birmingham, Birmingham, B15 2TT, UK; 2 CR UK Clinical Trials Unit, Institute for Cancer Studies, University of Birmingham, Birmingham, B15 2TT, UK; 3 Department of Surgery, Russells Hall Hospital, Pensett, Dudley, West Midlands, DY1 2HQ, UK

## Abstract

Evidence has shown that physical activity may attenuate the negative physical, psychological and functional effects of treatment in women diagnosed with breast cancer. Physical activity levels also decline substantially during and after completion of treatment for cancer, highlighting the importance of strategies to promote participation in regular physical activity in this population. Oncologists and surgeons may serve as an influential source of motivation to be physically activity in cancer patients, by conveying the importance of a healthy lifestyle. The primary purpose of the present study was to investigate whether oncologists and surgeons routinely discuss physical activity with their breast cancer patients and to investigate the nature of any information/advice provided during consultations. A secondary aim was to examine whether physically active oncologists and surgeons were more likely to provide advice about physical activity to patients, than inactive oncologists and surgeons. A brief postal questionnaire was sent to 710 consultant breast cancer oncologists and surgeons throughout the UK and 102 responded (response rate = 14.4%). Of responders, most (55.9%) did not routinely discuss physical activity with their patients. Amongst oncologists/surgeons (clinicians) who did offer advice, most focussed on discussing the benefits of physical activity for physical and functional health gains and for facilitating weight control and maintenance. A number of clinicians indicated they advised patients that physical activity may decrease risk of recurrence and improve survival, despite the lack of evidence from RCTs to support this suggestion. There was no significant association between the physical activity status of oncologists/surgeons and the likelihood that they discussed physical activity with patients. Educational strategies aimed at encouraging clinicians to promote physical activity in consultations need to be targeted widely amongst the cancer clinician community.

## Background

Evidence from RCTs has shown that physical activity may attenuate the negative effects of cancer treatment in women diagnosed with breast cancer [1,2]. Rates of physical activity decline substantially during cancer treatment and may not return to pre-diagnosis levels after treatment has been completed [3]. Recent epidemiological studies [4] have also demonstrated an inverse relationship between physical activity after diagnosis and risk of recurrence and breast cancer specific mortality, so the importance of maintaining a physically active lifestyle after diagnosis of breast cancer may have even greater health consequences.

Oncologists and surgeons may serve as an important source of motivation by encouraging patients to be physically active and by conveying the importance of a healthy lifestyle after cancer diagnosis. Oncologists have also been found to have a favourable attitude towards promoting exercise with cancer patients [5,6]. In addition, a recent well conducted RCT [7] demonstrated that a brief oncologist prompt to exercise during treatment consultations increased physical activity in newly diagnosed breast cancer patients, further highlighting the instrumental 'gatekeeper' role that cancer clinicians can have in facilitating changes in cancer patients' health behaviours.

Studies [5,6] conducted in North America have investigated whether oncologists provide physical activity advice to their patients, although little is known about which speciality of cancer clinician are likely to provide advice or the nature of the information provided. The primary aims of the present study were to investigate whether oncologists and surgeons in the UK routinely discussed physical activity with their breast cancer patients, to explore the nature of this advice and to investigate whether oncologists and surgeons who were physically active themselves, were more likely to discuss physical activity with their patients.

## Methods

As part of the development procedures for a RCT of the effects of physical activity on breast outcome, a brief anonymous postal questionnaire (see Appendix 1) was sent (one mailing with no reminders) to 710 consultants registered with the Cancer Research UK Clinical Trials Unit database and comprised of 332 surgeons, 255 clinical oncologists and 84 medical oncologists (clinicians). Clinicians were asked to report whether they routinely provided advice to patients about physical activity during consultations and to indicate the nature and context of any advice provided. An open comment section was included in the questionnaire where clinicians were given the opportunity to provide written details of the advice they gave to patients. Clinicians were also asked to indicate who they believed would be the most suitable health professional to deliver physical activity intervention/advice to breast cancer patients (i.e. nurse, surgeon, oncologist, physiotherapist or other health professional). In addition, the questionnaire also included items to assess clinicians' age, gender, medical speciality and the amount of moderate intensity physical activity that they typically achieved per week.

## Results

A total of 102 breast cancer consultants (medical oncologists: n = 14; clinical oncologists: n = 48; surgeons: n = 40) working across 65 sites in the UK responded to the study questionnaire (response rate = 14.4%). The majority of responders were from district hospitals (56%) and worked in specialist cancer centres (63%). Most respondents were aged 40–50 years (n = 44) or 50–60 years (n = 37). Of responders, 44.1% (n = 45) routinely gave advice to their patients about physical activity. Walking was the most commonly advocated type of activity although advice relating to the duration and intensity of physical activity that patients were encouraged to achieve varied considerably [see additional file]. The nature of the advice provided can be broadly categorised into five main themes; benefits for recurrence and mortality, benefits for weight control and management, benefits for physical and functional health, benefits of active healthy living and general comments about physical activity prescription [see additional file 1].

Chi-squared analysis showed that oncologists were significantly (p < 0.01) more likely to give patients advice about physical activity than surgeons (n = 36/60: 60.0% versus n = 9/38: 23.7%). Relatively few clinicians (36.5%: n = 33/85) were themselves meeting current public health recommendations for physical activity of at least 150 minutes of moderate intensity physical activity per week. Chi-squared analysis indicated no relationship between physical activity status (meeting the current physical activity recommendations or not) and whether clinicians promoted physical activity with their patients (yes or no). Clinicians felt nurses (50%: n = 51) and physiotherapists (33.3%: n = 34) were the most suitable health professionals to deliver physical activity interventions to breast cancer patients; 11.8% indicated 'other health professional' in response to this question (e.g. fitness instructor). Only 1.9% (n = 2) of clinicians felt oncologists were the most suitable person to deliver physical activity interventions and no respondents indicated that surgeons were suitable.

## Discussion

This study found that about 44% of clinicians gave advice to their patients about physical activity. This finding is consistent with previous research [6] conducted in North America which found 43% of oncologists reported that they tried to recommend physical activity to their patients, when appropriate. Related to this, a previous study [5] showed that about 28% of cancer patients reported that their oncologist discussed exercise during treatment consultations. While at face value findings may be considered encouraging, less than half of clinicians recommend physical activity to their patients. Oncologists and surgeons in the UK do not receive formal training on health behaviour issues and greater efforts need to be made to educate them about the important role of exercise in cancer care so that it becomes part of routine practice for all clinicians. This is also important because evidence shows that cancer patients are likely to be receptive to advice about exercise from clinicians [8] and because [5] cancer patients who report that their oncologist discussed exercise during treatment consultations report higher levels of exercise during subsequent treatment.

Oncologists were much more likely to promote physical activity with their patients than surgeons; this might be because oncologists have greater levels of contact with patients during follow-up and/or because they see patients at the completion of treatment and give formal advice on prevention of relapse. In contrast, surgeons see patients during active treatment when advice about physical activity may not be as appropriate. These findings also serve to highlight differences in practice between clinicians of different sub-specialities and help to identify where the greatest need for training might lie. Results are also in broad agreement with a previous study [6] which found oncologists had favourable opinions about promoting exercise with their patients.

Amongst clinicians who did offer physical activity advice, most focussed on the physical and functional benefits that it can provide and its role in facilitating weight maintenance. It was interesting to note that many clinicians discussed physical activity with patients in the context that participation may decrease their risk of recurrence and improve survival. Currently there is only epidemiological evidence to support an association between physical activity and mortality from breast cancer and RCTs are still required to confirm any potential survival benefit.

About a third of clinicians were meeting the current public health recommendations for physical activity per week, which is similar to rates found for the general population in the UK [9]. Those clinicians who were active themselves were no more likely than their inactive counterparts to promote physical activity with patients, suggesting that any educational strategies aimed at encouraging clinicians to promote physical activity in consultations need to be targeted widely amongst the cancer clinician community. It was also interesting to note that very few clinicians felt that they were best placed to offer patients advice about physical activity, yet many were routinely doing so.

This study should be interpreted in the context of several limitations. The response rate was low and those who did respond may not be representative of all oncologists and surgeons. Larger studies may be better positioned to explore these issues more precisely. However, responses were obtained from male and female clinicians of different ages located at sixty-five sites throughout the UK and this should serve to increase the generalisability of the findings. It is anticipated that responders were more likely to have some interest in physical activity than non-responders and therefore these findings may represent a 'best case scenario' regarding current practice of cancer clinicians.

To the best of our knowledge this is the first study of its kind to take place in the UK and to provide information about both the qualitative and quantitative nature of the physical activity advice given to patients by oncologists and surgeons. Previous studies from North America have focussed on oncologists and less is known about the practice of cancer surgeons in relation to physical activity; the present study addresses this gap in the literature.

## Conclusion

Many clinicians discuss physical activity with their patients but a large proportion do not. Few clinicians felt that they were best placed to offer patients advice about physical activity and this may explain why many do not do so. Greater efforts need to be made to educate cancer clinicians in the UK about physical activity so that patients are routinely advised about the health benefits that participation might provide both during and after active treatment for breast cancer.

## List of abbreviations

RCTs: randomised controlled trials; UK: United Kingdom.

## Competing interests

The authors declare that they have no competing interests.

## Authors' contributions

AJD drafted the manuscript and conducted the analyses. SJB coordinated the study, helped to draft the manuscript and contributed to data analysis. All authors participated in conceptualisation and design of the questionnaire study. All authors read and approved the final version of the manuscript.

## Appendix 1

### Study Questionnaire

1) Indicate your profession:

Clinical Oncologist □ Medical Oncologist □ Surgeon □

2) Would you normally give patients advice regarding exercise? No □ Yes □

If yes, briefly describe the advice you would give below:

_______________________________________________________________________________________

_______________________________________________________________________________________

_______________________________________________________________________________________

3) Who would you consider most suitable to deliver an exercise intervention?

Research Nurse □ Physiotherapist □

Surgeon □ Oncologist □

Other □ please specify:_____________________________

4) We would be grateful if you could provide the following optional information about yourself

a) What is your gender? Female □ Male □

b) What is your age range in years? < 30 □ 30–40 □ 40–50 □ 50–60 □ > 60 □

c) In a typical week how many times, and for how long, do you participate in moderate intensity exercise (e.g. fast walking, cycling, swimming, jogging, aerobics)

Number of times per week: _____________

Average time per session: _____________ minutes

## Supplementary Material

Additional file 1 Advice given to breast cancer patients by consultant oncologists and surgeons in the UK. This information provided an overview of the type of advice given to breast cancer patients by consultant oncologists and surgeons. Click here for file
