# Supplementary material for: What advice are oncologists and surgeons in the United Kingdom giving to breast cancer patients about physical activity?
Source: Int J Behav Nutr Phys Act. 2008 Sep 19;5:46. doi: 10.1186/1479-5868-5-46 (PMC2553795; doi:10.1186/1479-5868-5-46)
Supplement: Additional file 1 — Advice given to breast cancer patients by consultant oncologists and surgeons in the UK. This information provided an overview of the type of advice given to breast cancer patients by consultant oncologists and surgeons. [file 1479-5868-5-46-S1.doc]

**Advice about benefits of physical activity for recurrence and mortality**

Moderate (as opposed to small amount of exercise) amount of exercise has been associated with better outcomes in breast cancer.

I am convinced that regular exercises is of benefit to all and advise patients that recovery from breast cancer is opportunity to take measures to improve lifestyle and also evidence that obesity increases risk of recurrence

That moderate exercise such as walking or swimming reduces the risk or recurrence of breast cancer

Explain that there is some evidence that regular exercise reduces risk of death

Advise that moderate exercise reduces risk of recurrence and advise 20 minutes of walking per day

**Advice about benefits of physical activity for weight control and management**

Tell them that evidence that exercise and weight control may have beneficial effect. Also refer them to breast cancer centre DVD on diet/exercise produced Jan '07

Advice about weight. Recommend at least 3 sessions of moderate aerobic activity per week, e.g. half an hour of walking or swimming

Advised to try to maintain stable weight during chemotherapy

Advice about weight. Recommend at least 3 sessions of moderate activity per week, e.g., half an hour walk or swim

General - risk of obesity & benefits of exercise

**Advice about benefits of physical activity for physical and functional health**

To prevent lymphoedema

To maintain normal activity and strengthen muscle pre and post op

Patients would be given general advice regarding maintaining physical fitness etc

Minimal general advice on walking - regularly - manageable exercise and good for osteoporosis

**Advice about the benefits of active healthy living**

If asked - try to gradually increase exercise. There is some evidence that patients who manage this do better

Good for you but don't push beyond your limits

Advise all patients that 'regular exercise shown to be beneficial'

Explain that healthy lifestyle has been shown to be beneficial and hence encourage exercise within their capabilities

Some times mention benefit of healthy living, particularly diet and exercise to all patients

Briefly encourage exercise as part of lifestyle advice along with diet etc. No specific advice given but encouraged to participate in regular exercise

**General advice about physical activity and exercise prescription**

For post-menopausal women 40 minutes daily walking

Regular exercise - walking/swimming (rather than jogging/weight training)

According to their level of fitness but advise regular walking

To continue to do all normal activities including exercising/ sport within their tolerance. If not usually physically active I suggest that they try to do some walking most days

Walking/jogging to increase from present level.

Increase activity in and around house.

Encourage daily aerobic exercise, e.g., walking dog, swimming as well as healthy (low fat, vegetables, fruit) diet.

As much as possible

Depending on the patient's PS and co morbid conditions - advise to do at least 5 hours of metabolic equivalent activity per week.

General advice to do some form of regular exercise

Important to return to normal physical activity early following surgery/therapy for breast cancer

To continue usual activities (if any!) after informing gym etc. of diagnosis as long as tolerates activity (during chemotherapy).

Regular weight bearing exercise half an hour 3-5 times/week especially if starting aromatase inhibitors.

Only to return to normal activity

Advised to exercise within their capabilities - no specific programme

Increase aerobic physical activity after recovery from adjuvant treatments. Gentle exercise during chemo/radiotherapy if patient feels able to.

Advocate gentle exercise on a daily basis

Aerobic exercise 30 minutes three times per week
